# Association Between Rewarming Rate and Short-term Outcomes After Repair of DeBakey Type I Acute Aortic Dissection: A Propensity Score-matched Analysis

**DOI:** 10.1093/icvts/ivag073

**Published:** 2026-03-04

**Authors:** Chao Deng, Hao Tang, Kangjun Shen, Ting Lu, Bo Jiang, Jingyu Li, Zhengxiong Li, Song Tian, Ling Tan

**Affiliations:** Department of Cardiovascular Surgery, The Second Xiangya Hospital of Central South University, Changsha 410011, Hunan, China; Department of Cardiovascular Surgery, The Second Xiangya Hospital of Central South University, Changsha 410011, Hunan, China; Department of Cardiovascular Surgery, The Second Xiangya Hospital of Central South University, Changsha 410011, Hunan, China; Department of Cardiovascular Surgery, The Second Xiangya Hospital of Central South University, Changsha 410011, Hunan, China; Department of Cardiovascular Surgery, The Second Xiangya Hospital of Central South University, Changsha 410011, Hunan, China; Department of Cardiovascular Surgery, The Second Xiangya Hospital of Central South University, Changsha 410011, Hunan, China; Department of Cardiovascular Surgery, The Second Xiangya Hospital of Central South University, Changsha 410011, Hunan, China; Department of Cardiovascular Surgery, The Second Xiangya Hospital of Central South University, Changsha 410011, Hunan, China; Department of Cardiovascular Surgery, The Second Xiangya Hospital of Central South University, Changsha 410011, Hunan, China

**Keywords:** rewarming rate, DeBakey type I acute aortic dissection, aortic arch surgery, short-term death

## Abstract

**Objectives:**

To evaluate the association between rewarming rate and short-term postoperative outcomes after repair of DeBakey type I acute aortic dissection (AAD).

**Methods:**

From January 2019 to November 2023, 763 patients with DeBakey type I AAD undergoing total arch replacement (TAR) and the frozen elephant trunk (FET) procedure were enrolled. Patients were categorized into 3 groups according to bladder rewarming rate: high (≥0.5°C/min), medium (0.2-0.5°C/min), and low (≤0.2°C/min). Propensity score matching (PSM) was applied to balance baseline characteristics, and short-term postoperative outcomes were compared across groups.

**Results:**

After PSM, the incidence of short-term death differed significantly across the 3 bladder rewarming rate groups (overall *P* value = .021). Pairwise comparisons showed a higher incidence of short-term death in the ≥0.5°C/min group compared with the 0.2-0.5°C/min group (Holm-adjusted *P* value = .041), whereas no significant differences were observed between other pairs. No significant differences were found in other major short-term postoperative outcomes.

**Conclusions:**

In patients with DeBakey type I AAD who undergo TAR and FET procedure, faster bladder rewarming (at ≥0.5°C/min) may increase the risk of short-term death, whereas slower rewarming (at ≤0.2°C/min) does not appear to adversely affect early postoperative outcomes. These findings should be interpreted with caution and underscore the need for further prospective investigation to define optimal rewarming strategies.

## Graphical Abstract

**Figure:**
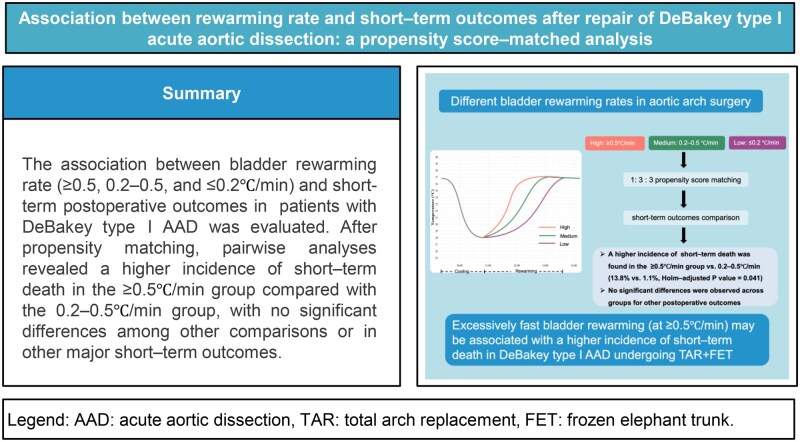

## INTRODUCTION

During aortic arch surgery, interruption of organ perfusion during cardiopulmonary bypass (CPB) can lead to ischaemic hypoxic injuries.^1^^,^^2^ Hypothermic circulatory arrest (HCA), a standard technique in aortic arch procedures, mitigates the risk of such injuries by slowing tissue metabolism and thereby protecting organs.^3^ However, temperature management during HCA, including the degree of hypothermia and the rates of cooling and rewarming,^4^ plays a crucial role in organ protection. Whereas slow rewarming can prolong CPB time, extend hypothermic exposure, and impair coagulation,^5^^,^^6^ rapid rewarming may disrupt the balance between oxygen demand and supply, potentially worsening organ function.^7^ According to clinical guidelines of the Society of Thoracic Surgeons, the Society of Cardiovascular Anesthesiologists, and the American Society of Extracorporeal Technology, the rewarming rate should be <0.5°C/min when arterial blood outlet temperature exceeds 30°C.^8^ However, temperature management during CPB, particularly for rewarming, remains controversial,^9^^,^^10^ and few relevant studies have focused on aortic arch surgery.

We retrospectively studied patients with DeBakey type I acute aortic dissection (AAD) who underwent total arch replacement (TAR) and frozen elephant trunk (FET) procedure to evaluate the association between bladder rewarming rate—categorized as high (≥ 0.5°C/min), medium (0.2-0.5°C/min), and low (≤0.2°C/min)—and short-term postoperative outcomes.

## METHODS

### Ethics statement

This retrospective study was approved by the Ethics Committee of the Second Xiangya Hospital of Central South University, Changsha, Hunan, China (LYF2023113), on August 2, 2023. The requirement for written informed consent was waived. All procedures were conducted in accordance with requirements outlined in the WMA Declaration of Taipei.

### Patient recruitment

**Figure 1.**
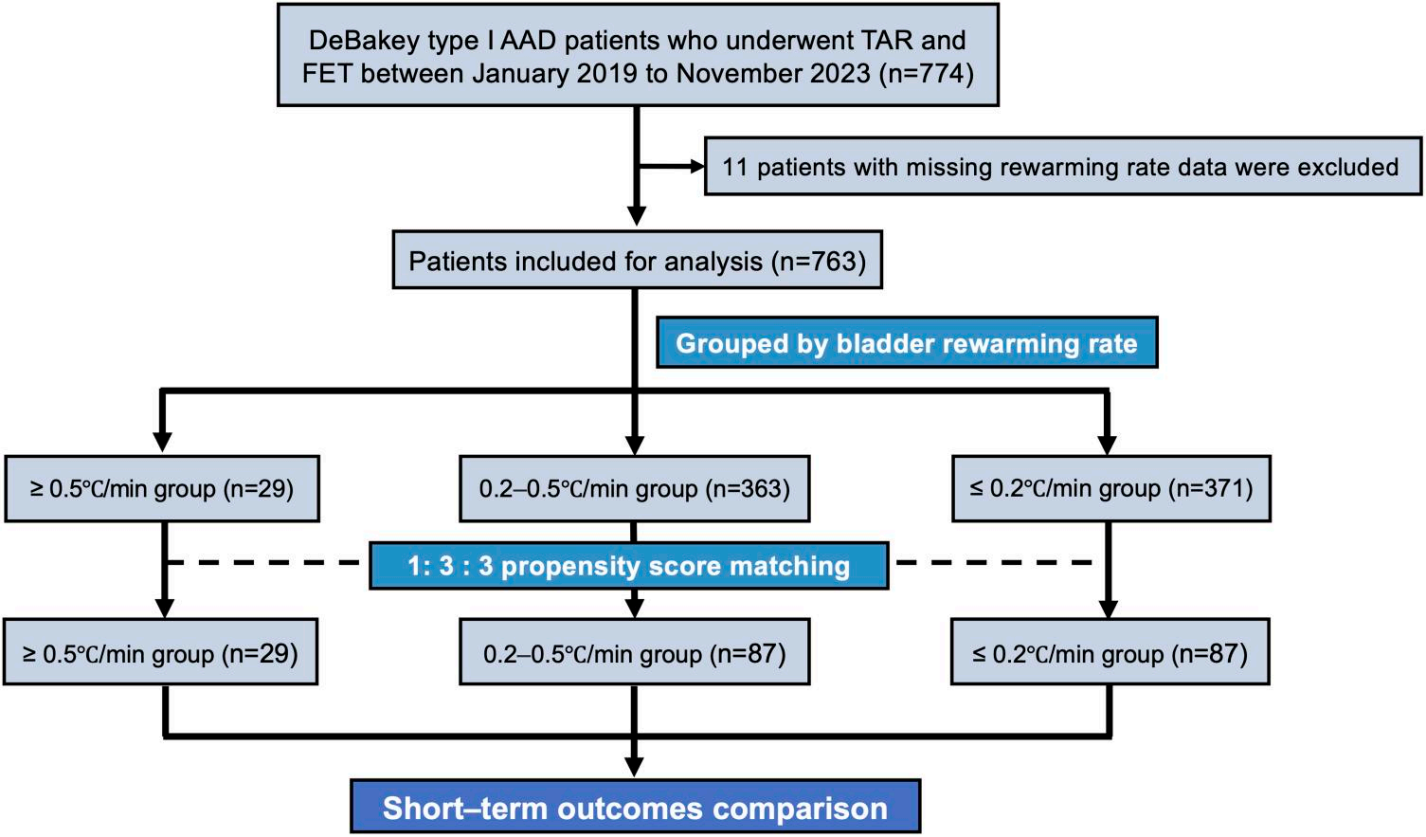
Flowchart of Patient Selection and Propensity Score Matching. The final population of this study was 763 patients with DeBakey type I acute aortic dissection (AAD) who underwent total arch replacement (TAR) and frozen elephant trunk (FET) procedure. Patients were categorized into 3 groups according to bladder rewarming rate: ≥0.5°C/min, 0.2-0.5°C/min, and ≤0.2°C/min. Propensity score matching in a 1:3:3 ratio was applied to balance baseline characteristics, and short-term postoperative outcomes were compared across groups

Between January 2019 and November 2023, 774 patients with DeBakey type I AAD who had undergone TAR and FET surgery at the Second Xiangya Hospital of Central South University were screened. Eleven patients were excluded from the study because rewarming rate data were missing; thus, the final sample included 763 patients. A flowchart of patient selection and propensity score matching (PSM) is illustrated in **Figure 1**.

### Surgical techniques

A 4-branched graft was used to reconstruct the aortic arch, and a 12-cm CRONUS stent graft (MicroPort Scientific Corp, Shanghai, China) was inserted into the true lumen of the descending aorta. The anastomosis consisted sequentially of the proximal ascending aorta, left carotid artery, proximal descending aorta, innominate artery, and left subclavian artery. The choice between moderate or mild hypothermia and of cerebral perfusion method (bilateral or unilateral) was made by the surgeon and depended on patient-specific considerations. Perfusion flow during lower body circulatory arrest (LBCA) was determined by the level of HCA (8-10 mL/kg/min or 0.6-1.2 L/m^2^/min). The surgical technique was based primarily on the approaches detailed previously.^11–13^

### Rewarming protocol

Rewarming was initiated once the jugular venous oxygen saturation (SjvO_2_) reached 80%. During rewarming, the temperature gradient between the patient and the heater-cooler unit was maintained at ≤5°C, and the water temperature was kept below 37°C.

### Definitions

Short-term death was defined as death from any cause within 30 days after surgery. Ischaemic stroke was diagnosed on the basis of following events: (1) new onset of symptoms within 30 days after surgery; (2) symptoms that included focal neurological dysfunctions, such as weakness or numbness of 1 side of the face or limbs, speech disorders, and other comprehensive neurological deficits; (3) unlimited duration of symptoms and signs when the responsible lesions were confirmed by computed tomography or magnetic resonance imaging; otherwise, the duration should be more than 24 hours; (4) exclusion of non-vascular causes of symptoms and signs; and (5) exclusion of postoperative haemorrhagic stroke. Cardiac arrest was defined as the sudden cessation of cardiac activity, causing the patient to become unresponsive, with no normal breathing and no signs of circulation^14^; however, episodes of cardiac arrest that occurred immediately before death were not included in this outcome category. Acute kidney injury (AKI) is defined according to the 2012 Kidney Disease | Improving Global Outcomes (KDIGO) clinical practice guidelines.^15^

### Statistical analyses

Patients were categorized into 3 groups according to bladder rewarming rate: high (≥ 0.5°C/min), medium (0.2-0.5°C/min), and low (≤ 0.2°C/min). The missing data (< 20%) were assumed to be missing at random and handled by multiple imputation using the Markov Chain Monte Carlo method with predictive mean matching. Five imputed datasets were generated with 10 iterations each. The imputation model included all preoperative variables, intraoperative variables, and postoperative maximal levels of serum creatinine, from which the incidence of AKI was subsequently determined (**Table S1**). Categorical variables were compared using the chi-square test, the corrected chi-square test, or Fisher’s exact test. Continuous variables were assessed using the 1-way ANOVA test (normal distributions) or the Kruskal-Wallis test (non-normal distributions). Pairwise comparisons were conducted for each outcome, with Holm adjustment across the 3 contrasts.

PSM was applied to minimize confounding,^16^ using a 1:3:3 ratio with nearest-neighbour matching for the logit of the propensity score. All patients in the high group (≥ 0.5°C/min) were retained as anchors, and strict nonreuse was enforced. Detailed grouping and matching were illustrated in **Figure 1**. Covariates for PSM analyses included age, sex, height, past medical history, and the following blood test results: white blood cell count, platelet count, haematocrit, international normalized ratio, activated partial thromboplastin time, alanine aminotransferase level, and creatinine level. Additional variables included left ventricular diameter and left ventricular ejection fraction; time from onset to admission and time from admission to operation; concomitant procedures (the Bentall procedure, aortic sinus repair, and coronary artery bypass grafting); cannulation methods; the durations of CPB, aorta clamping, and LBCA; repeated CPB; LBCA flow; and nasopharyngeal/bladder temperature during LBCA. Balance after matching was assessed using pairwise standardized mean differences together with an overall 3-group *P* value for each covariate, using the same testing strategy for categorical and continuous variables.

Two types of sensitivity analyses were performed. First, for the matched cohort, Kaplan-Meier survival curves were constructed for the 3 patient groups and compared them by using the log-rank test; pairwise log-rank comparisons were additionally conducted with Holm adjustment across the 3 contrasts. Second, for the overall cohort, a multivariable generalized linear model (binomial family) was used to evaluate the association between bladder rewarming rate (as the same 3-level categorical variable) and short-term death (candidate covariates were those that in univariable screening with a *P* value < .1, **Table S2**). Prespecified subgroup analyses were conducted across age (≥50 years vs < 50 years), sex, hypertension history, circulatory arrest temperature (mild hypothermia vs non-mild hypothermia), and cannulation methods (axillary, femoral, combined axillary and femoral, or other). Interaction terms were included in generalized linear models. Wald tests evaluated within-subgroup effects, and likelihood ratio tests assessed interaction effects; for CPB duration, interaction was evaluated by modelling CPB as a continuous variable. Subgroup analyses were conducted using “TableSubgroupMultiGLM” packages in RStudio 4.4.1, and multiple imputation and other analyses were performed in SPSS 27.0. All *P* values were 2-tailed, and a value of *P* < .05 was considered statistically significant.

## RESULTS

### Patients’ characteristics

Data from 763 patients were analysed. Baseline characteristics are summarized in **Table S3**. The overall incidences of short-term death and cardiac arrest were 5.8% (*n* = 44) and 5.4% (*n* = 41), respectively, and ischaemic stroke occurred in 49 patients (6.4%). Patients were stratified into 3 groups based on bladder rewarming rate: ≥0.5°C/min (*n* = 29, 3.8%), 0.2-0.5°C/min (*n* = 363, 47.6%), and ≤0.2°C/min (*n* = 371, 48.6%). After 1:3:3 PSM, the matched cohort comprised 203 patients, including 29 with a bladder rewarming rate ≥0.5°C/min, 87 with 0.2-0.5°C/min, and 87 with ≤0.2°C/min (**Figure 1**). No significant differences in preoperative or intraoperative variables were observed between the matched groups, and overall covariate balance was satisfactory (**Table  1**). Propensity-score distribution plots showed adequate overlap between the matched groups (**Figure S1**).

**Table 1.** Preoperative and Intraoperative Variables of Matched Patients Grouped by Bladder Rewarming Rate

Variables	All matched (*n *= 203)	Bladder rewarming rate groups			SMD			*P* value
		≥0.5°C/min (*n* = 29)	0.2-0.5°C/min (*n* = 87)	≤0.2°C/min (*n* = 87)	≥0.5°C/min vs. 0.2-0.5°C/min	≥0.5°C/min vs. ≤0.2°C/min	≤0.2°C/min vs. 0.2-0.5°C/min	
Male	144 (70.9%)	22 (75.9%)	57 (65.5%)	65 (74.7%)	0.227	0.027	0.201	.336
Age (years)	47.00 (11.34)	46.10 (11.52)	48.56 (10.28)	45.74 (12.19)	0.228	0.031	0.252	.233
Height (cm)	167.68 (8.71)	167.97 (9.07)	167.14 (9.01)	168.13 (8.35)	0.093	0.019	0.114	.744
Hypertension	163 (80.3%)	23 (79.3%)	70 (80.5%)	70 (80.5%)	0.029	0.029	0.000	.990
CAD	9 (4.4%)	1 (3.4%)	4 (4.6%)	4 (4.6%)	0.058	0.058	0.000	1.000
Previous heart surgery	11 (5.4%)	3 (10.3%)	3 (3.4%)	5 (5.7%)	0.272	0.169	0.110	.332
Diabetes mellitus	20 (9.9%)	4 (13.8%)	8 (9.2%)	8 (9.2%)	0.144	0.144	0.000	.689
Smoking	72 (35.5%)	10 (34.5%)	31 (35.6%)	31 (35.6%)	0.024	0.024	0.000	.993
White blood cell count (10^9/L)	12.94 (3.50)	13.14 (3.60)	12.83 (3.67)	12.99 (3.34)	0.084	0.043	0.044	.912
Haematocrit (%)	39.61 (5.45)	40.03 (6.14)	39.35 (5.23)	39.73 (5.47)	0.121	0.052	0.072	.813
Platelet count (10^9/L)	192.11 (70.87)	200.55 (88.91)	188.72 (67.72)	192.69 (67.84)	0.152	0.101	0.059	.737
INR	1.08 (0.18)	1.12 (0.26)	1.07 (0.16)	1.08 (0.16)	0.246	0.189	0.074	.390
APTT (seconds)	35.30 (6.96)	36.19 (6.67)	35.44 (7.53)	34.87 (6.48)	0.106	0.204	0.083	.654
Alanine transaminase (U/L)	23.70 [15.10, 34.20]	23.50 [18.70, 37.90]	22.90 [18.40, 35.90]	22.30 [17.60, 42.65]	0.035	0.094	0.049	.377
Creatinine (μmol/L)	154.75 (188.35)	174.20 (289.64)	129.84 (133.68)	173.17 (192.48)	0.200	0.004	0.263	.265
Left ventricle diameter (cm)	47.12 (5.56)	47.24 (5.02)	47.45 (5.57)	46.76 (5.77)	0.039	0.090	0.122	.712
LVEF (%)	63.84 (8.74)	63.83 (6.46)	64.16 (9.88)	63.52 (8.24)	0.040	0.042	0.071	.890
Time from onset to admission (hours)	12.00 [8.00, 22.00]	13.00 [10.00, 24.00]	14.00 [10.00, 24.50]	11.00 [7.00, 19.00]	0.057	0.142	0.092	.035
Time from admission to operation (hours)	14.00 [8.25, 21.83]	13.08 [10.00, 19.33]	13.66 [7.79, 21.00]	14.50 [8.12, 22.00]	0.154	0.026	0.115	.805
Concomitant procedures								
Bentall	11 (5.4%)	2 (6.9%)	5 (5.7%)	4 (4.6%)	0.047	0.099	0.052	.914
Aortic sinus repair	135 (66.5%)	18 (62.1%)	62 (71.3%)	55 (63.2%)	0.195	0.024	0.171	.458
CABG	10 (4.9%)	1 (3.4%)	4 (4.6%)	5 (5.7%)	0.058	0.110	0.052	1.000
Cannulation					0.069	0.140	0.148	.946
Axillary artery	119 (58.6%)	16 (55.2%)	49 (56.3%)	54 (62.1%)				
Femoral artery	13 (6.4%)	2 (6.9%)	5 (5.7%)	6 (6.9%)				
Axillary and femoral arteries	66 (32.5%)	10 (34.5%)	31 (35.6%)	25 (28.7%)				
Others	5 (2.5%)	1 (3.4%)	2 (2.3%)	2 (2.3%)				
Durations (min)								
CPB	245.49 (84.02)	252.34 (90.78)	246.86 (86.45)	241.84 (79.95)	0.063	0.124	0.061	.828
Aorta clamping	118.73 (51.64)	120.45 (56.88)	122.08 (47.60)	114.82 (54.01)	0.032	0.103	0.144	.640
LBCA	24.42 (9.15)	24.97 (9.85)	24.67 (9.73)	23.98 (8.37)	0.030	0.110	0.077	.831
Repeated CPB	50 (24.6%)	7 (24.1%)	19 (21.8%)	24 (27.6%)	0.055	0.079	0.133	.678
Flow of LBCA (L/min)	1.32 (0.67)	1.32 (0.66)	1.33 (0.71)	1.31 (0.64)	0.016	0.025	0.040	.966
Bladder temperature (°C)	28.22 (2.06)	28.11 (2.32)	28.15 (2.17)	28.32 (1.87)	0.015	0.098	0.084	.829

Abbreviations: APTT, activated partial thromboplastin time; CABG, coronary artery bypass grafting; CAD, coronary artery disease; CPB, cardiopulmonary bypass; INR, international normalized ratio; LBCA, lower body circulatory arrest; LVEF, left ventricular ejection fraction; SMD, standard mean difference.

### Postoperative outcomes

Postoperative outcomes among the matched patients grouped by bladder rewarming rate were summarized in **Table 2**. After PSM, the incidence of short-term death differed significantly across the 3 bladder rewarming rate groups (overall *P* value = .021). Holm-adjusted pairwise comparisons revealed the incidence of short-term death was higher in the ≥0.5°C/min group than in the 0.2-0.5°C/min group (13.8% vs 1.1%, Holm-adjusted *P* value = .041). The incidence of short-term death did not differ significantly between the ≤0.2°C/min group and 0.2-0.5°C/min group (5.7% vs 1.1%) or between the ≥0.5°C/min group and ≤0.2°C/min group (13.8% vs 5.7%) (both Holm-adjusted *P* value = .421). No significant differences were observed across bladder rewarming rate groups in other postoperative outcomes (**Table 2**). In addition, the nasopharyngeal rewarming rate was analysed independently, but no significant associations with postoperative outcomes were observed (**Tables S4-S6**).

**Table 2.** Outcomes of Matched Patients Grouped by Bladder Rewarming Rate

Variables	All matched (*n* = 203)	Bladder rewarming rate groups			Overall *P* value	Pairwise Holm-adjusted *P* value		
		≥0.5°C/min (*n* = 29)	0.2-0.5°C/min (*n* = 87)	≤0.2°C/min (*n* = 87)		≥0.5°C/min vs. 0.2-0.5°C/min	≥0.5°C/min vs. ≤0.2°C/min	≤0.2°C/min vs. 0.2-0.5°C/min
Short-term death	10 (4.9%)	4 (13.8%)	1 (1.1%)	5 (5.7%)	.021	0.041	0.421	0.421
Ischaemic stroke	9 (4.4%)	0 (0.0%)	5 (5.7%)	4 (4.6%)	.583	0.988	1.000	1.000
AKI	92 (45.3%)	12 (41.4%)	40 (46.0%)	40 (46.0%)	.899	1.000	1.000	1.000
CRRT	30 (14.8%)	7 (24.1%)	7 (8.0%)	16 (18.4%)	.040	0.127	0.501	0.127
Paraplegia	5 (2.5%)	1 (3.4%)	1 (1.1%)	3 (3.4%)	.561	1.000	1.000	1.000
Cardiac arrest	9 (4.4%)	4 (13.8%)	2 (2.3%)	3 (3.4%)	.043	0.101	0.129	1.000
Tracheostomy	2 (1.0%)	0 (0.0%)	1 (1.1%)	1 (1.1%)	1.000	1.000	1.000	1.000
ICU duration (days)	7.25 (11.23)	6.17 (5.64)	7.87 (15.10)	6.99 (7.54)	.749	1.000	1.000	1.000

Abbreviations: AKI, acute kidney injury; CRRT, continuous renal replacement therapy; ICU, intensive care unit.

### Sensitivity and subgroup analyses

**Figure 2.**
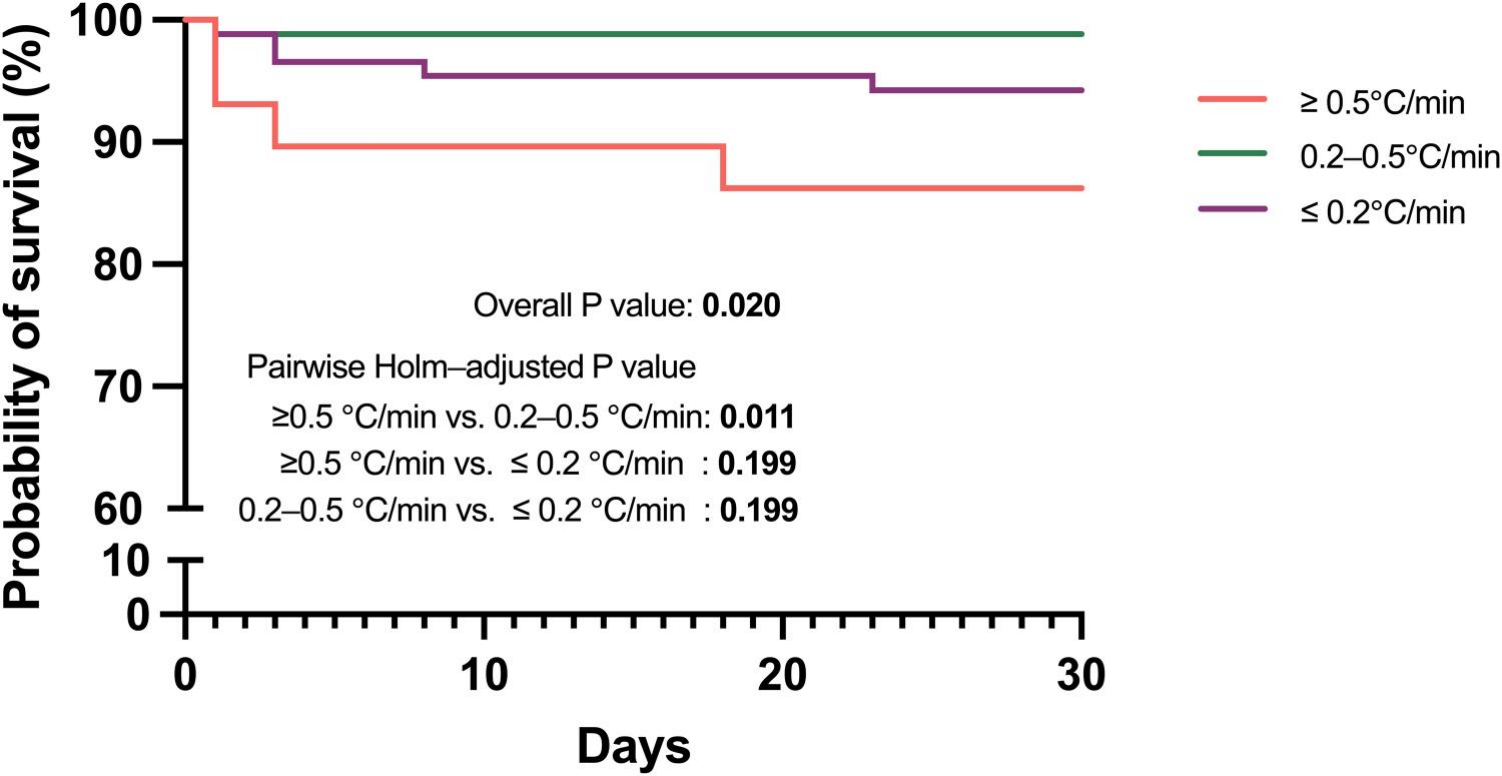
Kaplan-Meier Survival Curves for Short-Term Death Group by Bladder Rewarming Rate in the Matched Cohort

For the matched cohort (*n* = 203), Kaplan-Meier survival curves were generated and compared using the log-rank test to evaluate differences in survival across bladder rewarming rate groups (**Figure 2**). Overall survival differed significantly among the 3 groups (overall *P* value = .02). Holm-adjusted pairwise comparisons revealed that the probability of survival was significantly lower in the ≥0.5°C/min group than the 0.2-0.5°C/min group (Holm-adjusted *P* value = .011), whereas no significant differences were observed between the other pairs (both Holm-adjusted *P* value = .199).

For the overall cohort (*n* = 763), multivariable generalized linear model revealed that the incidence of short-term death was higher in the ≥0.5°C/min group than the 0.2-0.5°C/min group (odds ratio 4.17; 95% confidence interval, 1.15-14.29; nominal *P* value = .030; **Figure S2**); however, this association was attenuated after adjustment for multiple pairwise comparisons (Holm-adjusted *P* value = .090). All interaction tests were non-significant (*P* value for interaction > .05), indicating no evidence of effect modification across age, sex, hypertension, circulatory arrest temperature, cannulation methods, or CPB duration.

## DISCUSSION

As an essential technique for organ protection in aortic arch surgery, the role of HCA continues to evolve.^17^ Deep HCA entails a safe ischaemic interval and a bloodless surgical field; however, it also extends the period needed for oxygen debt repayment and rewarming.^18^ Recently, a “warmer” temperature management approach has emerged, wherein moderate and even mild hypothermia is combined with antegrade cerebral perfusion, and the outcomes have been favourable.^2^^,^^9^^,^^19–22^ During rewarming, the rising metabolic demand must be matched by sufficient amounts of oxygen. An inappropriate rewarming process may lead to an imbalance between tissue metabolism and perfusion.^4^ In clinical practice guidelines, a rewarming rate below 0.5°C/min is recommended when the arterial blood outlet temperature exceeds 30°C; however, these guidelines offer no recommendation regarding a lower limit.^8^

For patients undergoing aortic arch surgery, our findings suggest that a bladder rewarming rate ≥ 0.5°C/min was not associated with increased incidences of neurological complications, AKI, cardiac arrest, or prolonged stay in the intensive care unit but was associated with a higher incidence of short-term death when compared with 0.2-0.5°C/min group. In contrast, the short-term outcomes of the ≤ 0.2°C/min group did not differ significantly when compared with either the 0.2-0.5°C/min group or ≥ 0.5°C/min group. These findings are consistent with previous findings of associations between fast rewarming and adverse postoperative outcomes. Grigore et al^7^ showed that postoperative cognitive function was better after slow rewarming (at temperatures increasing by 0.49°C ± 0.17°C/min) than after rapid rewarming (at temperatures increasing by 0.56°C ± 0.22°C/min). Similarly, Hori et al^23^ reported a link between faster rewarming and elevated levels of glial fibrillary acidic protein, a marker of brain injury. Linardi et al^5^ demonstrated an increase in inflammatory markers in rat hippocampal tissue under a fast-rewarming protocol. Furthermore, Saleh and Barr^24^ observed that among paediatric patients with congenital heart disease, cardiac performance (cardiac index and peak velocity) was best after the slowest rewarming, and the blood lactate levels were lowest. According to the 2024 aortic disease guidelines, keeping temperature gradients between the arterial and venous blood at <3°C, with temperature increases of <0.2°C/min, may reduce the incidence of AKI.^25^ Together, our findings support the notion that excessively fast rewarming (at ≥0.5°C/min) may be associated with adverse short-term outcomes, whereas slower rewarming (at ≤ 0.2°C/min) was not associated with worse postoperative outcomes and appears feasible in the context of aortic arch surgery.

In this retrospective study, the differences in rewarming rates between groups did not result from intentional regulation. Because rewarming was initiated only when SjvO_2_ reached 80%, the longer CPB duration in the high bladder rewarming rate group implies that SjvO_2_ remained suboptimal longer, even after the heart and left common carotid artery were perfused. Such prolonged suboptimal venous oxygenation indicates intraoperative physiologic derangement. We accordingly interpret fast rewarming as a marker of intraoperative instability. Physiologically, the rewarming rate may reflect what we refer to as “effective perfusion flow.” The operating-room temperature, temperature gradients, and perfusion flow (indexed according to body weight/body surface area) during CPB rewarming were controlled; therefore, rewarming rates may have been determined by regional circulation factors, such as effective circulating blood volume and peripheral vascular resistance. For example, local vasoconstriction reduces peripheral heat loss, causing the core temperature to rise faster and thereby worsening microcirculatory ischaemia,^26^ which is accompanied by an imbalance between effective perfusion flow and tissue metabolism.^4^ This may explain the link between fast rewarming and adverse outcomes. Moreover, excessive temperature gradients (particularly ≥10°C) may promote the release of dissolved gases from the blood, leading to microbubble formation.^8^ These gas emboli may enter the coronary microcirculation, impairing myocardial perfusion and increasing the risk for arrhythmias.^27^ In addition, bladder temperature measurements may reflect core temperature better than do nasopharyngeal measurements^25^; this possibility could explain why significant results were observed for bladder rewarming rates but not for nasopharyngeal rewarming rates. However, the process of rapid rewarming is often overlooked because rewarming rates are not monitored closely. In clinical practice, outcomes may be improved by vigilant temperature monitoring and real-time adjustments to temperature gradients or perfusion flow whenever rapid rewarming is detected.

This study had several limitations. First, selection bias was unavoidable in a single-centre retrospective analysis. Second, although baseline differences between the matched groups were controlled through PSM, number of patients undergoing fast rewarming may have been small enough to introduce bias. Third, some critical data, such as the full SjvO_2_ record during CPB, were unavailable due to the retrospective design.

## CONCLUSIONS

This single-centre retrospective study showed that bladder rewarming rate was a potentially important intraoperative factor associated with early outcomes in patients with DeBakey type I AAD who underwent TAR and FET procedure. Our findings suggested that excessively fast rewarming (at ≥ 0.5°C/min) may be associated with a higher risk of short-term death, whereas more conservative rewarming strategies (at ≤ 0.2°C/min) do not appear to adversely affect short-term postoperative outcomes. Although these findings should be interpreted with caution, they underscore the need to better define optimal rewarming practices in further prospective investigation.

## Supplementary Material

ivag073_Supplementary_Data

## Data Availability

The original data in this article are not publicly available due to privacy concerns and ethical restrictions. However, the data can be obtained from the corresponding author upon reasonable request.

## ABBREVIATIONS

AAD: acute aortic dissection
AKI: acute kidney injury
CI: confidence interval
CPB: cardiopulmonary bypass
FET: frozen elephant trunk
HCA: hypothermic circulatory arrest
LBCA: lower body circulatory arrest
OR: odds ratio
PSM: propensity score matching
SjvO₂: jugular venous oxygen saturation
TAR: total arch replacement
