# Supplementary material for: Association Between Rewarming Rate and Short-term Outcomes After Repair of DeBakey Type I Acute Aortic Dissection: A Propensity Score-matched Analysis
Source: Interdiscip Cardiovasc Thorac Surg. 2026 Mar 4;41(3):ivag073. doi: 10.1093/icvts/ivag073 (PMC13004222; doi:10.1093/icvts/ivag073)
Supplement: ivag073_Supplementary_Data [file ivag073_supplementary_data.zip › Supplementary Materials.docx]

**Supplementary Table S1. Preoperative and intraoperative conditions of unmatched patients before and after filling**

| **Variables** | **Before filling** | **After filling** | **Missing value**  **n (%)** |
| --- | --- | --- | --- |
| Male | 583 (76.4%) | 583 (76.4%) | 0 (0%) |
| Age (years) | 50.9 (11.1) | 50.9 (11.1) | 0 (0%) |
| Height (cm) | 167.1 (7.69) | 167.1 (7.69) | 0 (0%) |
| Hypertension | 556 (72.9%) | 556 (72.9%) | 0 (0%) |
| CAD | 42 (5.50%) | 42 (5.50%) | 0 (0%) |
| Previous heart surgery | 14 (1.83%) | 14 (1.83%) | 0 (0%) |
| Diabetes mellitus | 27 (3.54%) | 27 (3.54%) | 0 (0%) |
| Smoking | 303 (39.7%) | 303 (39.7%) | 0 (0%) |
| White blood cell count (10^9/L) | 12.7 (3.76) | 12.7 (3.76) | 0 (0%) |
| Hematocrit (%) | 39.2 (5.26) | 39.2 (5.26) | 0 (0%) |
| Platelet count (10^9/L) | 180.5 (69.4) | 180.5 (69.4) | 0 (0%) |
| INR | 1.10 (0.19) | 1.10 (0.19) | 0 (0%) |
| APTT (seconds) | 37.1 (7.22) | 37.1 (7.22) | 0 (0%) |
| Alanine transaminase (U/L) | 21.7 [14.0, 35.2] | 21.7 [14.0, 35.2] | 0 (0%) |
| Creatinine (μmol/L) | 120.7 (110.0) | 120.7 (110.0) | 0 (0%) |
| Left ventricle diameter (cm) | 46.9 (6.02) | 46.9 (6.01) | 1 (0.13%) |
| LVEF (%) | 65.1 (7.11) | 65.1 (7.11) | 1 (0.13%) |
| Time from onset to admission (hours) | 13.0 [8.00, 24.0] | 13.0 [8.00, 24.0] | 0 (0%) |
| Time from admission to operation (hours) | 16.0 [10.0, 28.0] | 16.0 [10.0, 28.0] | 0 (0%) |
| Concomitant procedures |  |  | 0 (0%) |
| Bentall | 52 (6.82%) | 52 (6.82%) |  |
| Aortic sinus repair | 401 (52.6%) | 401 (52.6%) |  |
| CABG | 48 (6.29%) | 48 (6.29%) |  |
| Cannulation |  |  | 0 (0%) |
| Axillary artery | 434 (56.9%) | 434 (56.9%) |  |
| Femoral artery | 115 (15.1%) | 115 (15.1%) |  |
| Axillary and femoral arteries | 200 (26.2%) | 200 (26.2%) |  |
| Others | 14 (1.83%) | 14 (1.83%) |  |
| Durations (minutes) |  |  |  |
| CPB | 232.6 (95.5) | 232.6 (95.5) | 0 (0%) |
| Aorta clamping | 110.0 (54.5) | 110.0 (54.5) | 0 (0%) |
| LBCA | 21.1 (7.75) | 21.1 (7.75) | 0 (0%) |
| Repeated CPB | 123 (16.1%) | 123 (16.1%) | 0 (0%) |
| Flow of LBCA (L/min) | 1.37 (0.64) | 1.36 (0.64) | 1 (0.13%) |
| Nasopharyngeal temperature (°C) | 27.8 (2.03) | 27.8 (2.03) | 0 (0%) |
| Bladder temperature (°C) | 28.6 (2.22) | 28.6 (2.22) | 0 (0%) |
| Nasopharyngeal rewarming rate (°C/min) | 0.30 (0.14) | 0.30 (0.14) | 0 (0%) |
| Bladder rewarming rate (°C/min) | 0.23 (0.12) | 0.23 (0.12) | 0 (0%) |
| Outcomes |  |  |  |
| Short–term death | 44 (5.77%) | 44 (5.77%) | 0 (0%) |
| Ischemic stroke | 49 (6.42%) | 49 (6.42%) | 0 (0%) |
| AKI | 292 (38.6%) | 296 (38.8%) | 6 (0.79%) |
| CRRT | 70 (9.17%) | 70 (9.17%) | 0 (0%) |
| Paraplegia | 14 (1.83%) | 14 (1.83%) | 0 (0%) |
| Cardiac arrest | 41 (5.37%) | 41 (5.37%) | 0 (0%) |
| Tracheostomy | 25 (3.28%) | 25 (3.28%) | 0 (0%) |
| ICU duration (days) | 6.89 (8.49) | 6.89 (8.49) | 0 (0%) |
| CI: confidence interval; CAD: coronary artery disease; INR: international normalized ratio; APTT: activated partial thromboplastin time; LVEF: left ventricular ejection fraction; CABG: coronary artery bypass grafting; CPB: cardiopulmonary bypass; LBCA: lower body circulatory arrest; AKI: acute kidney injury; CRRT: continuous renal replacement therapy; ICU: intensive care unit. | | | |

**Supplementary Table S2. Univariate analyses of short–term death for overall unmatched patients**

| **Variables** | **Overall (n = 763)** | **Mortality group (n = 44)** | **Non–mortality group (n = 719)** | **P value** |
| --- | --- | --- | --- | --- |
| Male | 583 (76.4%) | 31 (70.5%) | 552 (76.8%) | 0.438 |
| Age (years) | 50.9 (11.1) | 52.0 (10.3) | 50.8 (11.2) | 0.486 |
| Height (cm) | 167.1 (7.69) | 167.1 (9.07) | 167.1 (7.61) | 0.984 |
| Hypertension | 556 (72.9%) | 36 (81.8%) | 520 (72.3%) | 0.230 |
| CAD | 42 (5.50%) | 3 (6.82%) | 39 (5.42%) | 0.728 |
| Previous heart surgery | 14 (1.83%) | 2 (4.55%) | 12 (1.67%) | 0.191 |
| Diabetes mellitus | 27 (3.54%) | 3 (6.82%) | 24 (3.34%) | 0.200 |
| Smoking | 303 (39.7%) | 23 (52.3%) | 280 (38.9%) | 0.111 |
| White blood cell count (10^9/L) | 12.7 (3.76) | 12.9 (4.21) | 12.7 (3.74) | 0.719 |
| Hematocrit (%) | 39.2 (5.26) | 38.6 (6.53) | 39.3 (5.18) | 0.523 |
| Platelet count (10^9/L) | 180.5 (69.4) | 164.3 (52.4) | 181.5 (70.2) | 0.043 |
| INR | 1.10 (0.19) | 1.14 (0.26) | 1.10 (0.19) | 0.321 |
| APTT (seconds) | 37.1 (7.22) | 36.8 (7.64) | 37.1 (7.20) | 0.816 |
| Alanine transaminase (U/L) | 21.7 [14.0, 35.2] | 28.3 [15.1, 39.2] | 21.4 [14.0, 35.0] | 0.505 |
| Creatinine (μmol/L) | 120.7 (110.0) | 131.4 (144.8) | 120.1 (107.6) | 0.613 |
| Left ventricle diameter (cm) | 46.9 (6.02) | 49.0 (6.11) | 46.7 (5.99) | 0.019 |
| LVEF (%) | 65.0 (7.11) | 65.0 (6.95) | 65.0 (7.13) | 0.981 |
| Time from onset to admission (hours) | 13.0 [8.00, 24.0] | 12.0 [8.75, 21.0] | 13.0 [8.00, 24.0] | 0.511 |
| Time from admission to operation (hours) | 16.0 [10.0, 28.0] | 14.8 [9.06, 26.1] | 16.0 [10.0, 28.0] | 0.345 |
| Concomitant procedures |  |  |  |  |
| Bentall | 52 (6.82%) | 5 (11.4%) | 47 (6.54%) | 0.214 |
| Aortic sinus repair | 401 (52.6%) | 15 (34.1%) | 386 (53.7%) | 0.018 |
| CABG | 48 (6.29%) | 6 (13.6%) | 42 (5.84%) | 0.051 |
| Cannulation |  |  |  | 0.097 |
| Axillary artery | 434 (56.9%) | 21 (47.7%) | 413 (57.4%) |  |
| Femoral artery | 115 (15.1%) | 11 (25.0%) | 104 (14.5%) |  |
| Axillary and femoral arteries | 200 (26.2%) | 10 (22.7%) | 190 (26.4%) |  |
| Others | 14 (1.83%) | 2 (4.55%) | 12 (1.67%) |  |
| Durations (minutes) |  |  |  |  |
| CPB | 232.6 (95.5) | 303.6 (170.2) | 228.2 (87.2) | 0.006 |
| Aorta clamping | 110.0 (54.5) | 132.8 (77.7) | 108.6 (52.5) | 0.048 |
| LBCA | 21.1 (7.75) | 20.4 (7.47) | 21.2 (7.77) | 0.496 |
| Repeated CPB | 123 (16.1%) | 17 (38.6%) | 106 (14.7%) | <0.001 |
| Flow of LBCA (L/min) | 1.36 (0.64) | 1.51 (0.65) | 1.36 (0.63) | 0.130 |
| Nasopharyngeal temperature (°C) | 27.8 (2.03) | 28.0 (2.27) | 27.7 (2.01) | 0.550 |
| Bladder temperature (°C) | 28.6 (2.22) | 28.8 (2.50) | 28.5 (2.21) | 0.554 |
| CI: confidence interval; CAD: coronary artery disease; INR: international normalized ratio; APTT: activated partial thromboplastin time; LVEF: left ventricular ejection fraction; CABG, coronary artery bypass grafting; CPB: cardiopulmonary bypass; LBCA, lower body circulatory arrest. | | | | |

**Supplementary Table S3. Overall characteristics of unmatched patients grouped by bladder rewarming rate**

| **Variables** | **Overall (n = 763)** | **Bladder rewarming rate groups** | | | **Overall P value** |
| --- | --- | --- | --- | --- | --- |
|  |  | **≥0.5°C/min (n = 29)** | **0.2–0.5°C/min (n = 363)** | **≤0.2°C/min (n = 371)** |  |
| Male | 583 (76.4%) | 22 (75.9%) | 264 (72.7%) | 297 (80.1%) | 0.065 |
| Age (years) | 50.89 (11.14) | 46.10 (11.52) | 52.35 (10.71) | 49.84 (11.32) | <0.001 |
| Height (cm) | 167.09 (7.69) | 167.97 (9.07) | 166.58 (8.04) | 167.51 (7.21) | 0.211 |
| Hypertension | 556 (72.9%) | 23 (79.3%) | 266 (73.3%) | 267 (72.0%) | 0.673 |
| CAD | 42 (5.5%) | 1 (3.4%) | 22 (6.1%) | 19 (5.1%) | 0.879 |
| Previous heart surgery | 14 (1.8%) | 3 (10.3%) | 3 (0.8%) | 8 (2.2%) | 0.007 |
| Diabetes mellitus | 27 (3.5%) | 4 (13.8%) | 11 (3.0%) | 12 (3.2%) | 0.029 |
| Smoking | 303 (39.7%) | 10 (34.5%) | 126 (34.7%) | 167 (45.0%) | 0.014 |
| White blood cell count (10^9/L) | 12.69 (3.76) | 13.14 (3.60) | 12.34 (3.80) | 13.00 (3.72) | 0.048 |
| Hematocrit (%) | 39.24 (5.26) | 40.03 (6.14) | 38.85 (5.13) | 39.56 (5.31) | 0.133 |
| Platelet count (10^9/L) | 180.49 (69.41) | 200.55 (88.91) | 177.50 (65.28) | 181.85 (71.51) | 0.198 |
| INR | 1.10 (0.19) | 1.12 (0.26) | 1.11 (0.21) | 1.10 (0.17) | 0.673 |
| APTT (seconds) | 37.06 (7.22) | 36.19 (6.67) | 37.33 (7.35) | 36.87 (7.14) | 0.553 |
| Alanine transaminase (U/L) | 21.70 [14.00, 35.20] | 24.50 [17.70, 38.80] | 21.00 [13.55, 34.35] | 22.40 [14.25, 36.65] | 0.164 |
| Creatinine (μmol/L) | 120.71 (109.99) | 174.20 (289.64) | 110.16 (79.33) | 126.86 (109.72) | 0.003 |
| Left ventricle diameter (cm) | 46.86 (6.02) | 47.24 (5.02) | 46.89 (6.05) | 46.80 (6.06) | 0.921 |
| LVEF (%) | 65.05 (7.11) | 63.83 (6.46) | 65.28 (7.50) | 64.92 (6.76) | 0.506 |
| Time from onset to admission (hours) | 13.00 [8.00, 24.00] | 13.00 [10.00, 24.00] | 14.00 [9.00, 31.50] | 12.00 [8.00, 21.00] | <0.001 |
| Time from admission to operation (hours) | 16.00 [10.00, 28.00] | 13.08 [10.00, 19.33] | 16.00 [10.00, 30.29] | 16.00 [10.00, 26.62] | 0.381 |
| Concomitant procedures |  |  |  |  |  |
| Bentall | 52 (6.8%) | 2 (6.9%) | 25 (6.9%) | 25 (6.7%) | 1.000 |
| Aortic sinus repair | 401 (52.6%) | 18 (62.1%) | 188 (51.8%) | 195 (52.6%) | 0.566 |
| CABG | 48 (6.3%) | 1 (3.4%) | 24 (6.6%) | 23 (6.2%) | 0.965 |
| Cannulation |  |  |  |  | 0.092 |
| Axillary artery | 434 (56.9%) | 16 (55.2%) | 188 (51.8%) | 230 (62.0%) |  |
| Femoral artery | 115 (15.1%) | 2 (6.9%) | 64 (17.6%) | 49 (13.2%) |  |
| Axillary and femoral arteries | 200 (26.2%) | 10 (34.5%) | 105 (28.9%) | 85 (22.9%) |  |
| Others | 14 (1.8%) | 1 (3.4%) | 6 (1.7%) | 7 (1.9%) |  |
| Durations (minutes) |  |  |  |  |  |
| CPB | 232.58 (95.46) | 252.34 (90.78) | 233.97 (95.51) | 229.69 (95.79) | 0.436 |
| Aorta clamping | 110.02 (54.48) | 120.45 (56.88) | 111.86 (52.52) | 107.41 (56.13) | 0.313 |
| LBCA | 21.14 (7.75) | 24.97 (9.85) | 21.20 (7.98) | 20.78 (7.27) | 0.019 |
| Repeated CPB | 123 (16.1%) | 7 (24.1%) | 57 (15.7%) | 59 (15.9%) | 0.486 |
| Flow of LBCA (L/min) | 1.36 (0.64) | 1.32 (0.66) | 1.32 (0.62) | 1.41 (0.65) | 0.165 |
| Bladder temperature (°C) | 28.56 (2.22) | 28.11 (2.32) | 28.29 (2.25) | 28.86 (2.16) | 0.002 |
| Outcomes |  |  |  |  |  |
| Short–term death | 44 (5.8%) | 4 (13.8%) | 17 (4.7%) | 23 (6.2%) | 0.105 |
| Ischemic stroke | 49 (6.4%) | 0 (0.0%) | 26 (7.2%) | 23 (6.2%) | 0.367 |
| AKI | 296 (38.8%) | 12 (41.4%) | 141 (38.8%) | 143 (38.5%) | 0.955 |
| CRRT | 70 (9.2%) | 7 (24.1%) | 25 (6.9%) | 38 (10.2%) | 0.009 |
| Paraplegia | 14 (1.8%) | 1 (3.4%) | 6 (1.7%) | 7 (1.9%) | 0.591 |
| Cardiac arrest | 41 (5.4%) | 4 (13.8%) | 15 (4.1%) | 22 (5.9%) | 0.066 |
| Tracheostomy | 25 (3.3%) | 0 (0.0%) | 12 (3.3%) | 13 (3.5%) | 0.938 |
| ICU duration (days) | 6.89 (8.49) | 6.17 (5.64) | 7.14 (9.72) | 6.70 (7.31) | 0.703 |
| SMD: standard mean difference; CAD: coronary artery disease; INR: international normalized ratio; APTT: activated partial thromboplastin time; LVEF: left ventricular ejection fraction; CABG: coronary artery bypass grafting; CPB: cardiopulmonary bypass; LBCA: lower body circulatory arrest; AKI: acute kidney injury; CRRT: continuous renal replacement therapy; ICU: intensive care unit. | | | | | |

**Supplementary Table S4. Overall characteristics of unmatched patients grouped by nasopharyngeal rewarming rate**

| **Variables** | **Overall (n = 763)** | **Nasopharyngeal rewarming rate groups** | | | **Overall P value** |
| --- | --- | --- | --- | --- | --- |
|  |  | **≥0.5°C/min (n = 53)** | **0.2–0.5°C/min (n = 562)** | **≤0.2°C/min (n = 148)** |  |
| Male | 583 (76.4%) | 37 (69.8%) | 425 (75.6%) | 121 (81.8%) | 0.148 |
| Age (years) | 50.89 (11.14) | 50.32 (12.39) | 51.30 (11.11) | 49.55 (10.73) | 0.218 |
| Height (cm) | 167.09 (7.69) | 166.32 (7.03) | 166.94 (7.83) | 167.91 (7.39) | 0.302 |
| Hypertension | 556 (72.9%) | 40 (75.5%) | 416 (74.0%) | 100 (67.6%) | 0.264 |
| CAD | 42 (5.5%) | 2 (3.8%) | 35 (6.2%) | 5 (3.4%) | 0.339 |
| Previous heart surgery | 14 (1.8%) | 3 (5.7%) | 8 (1.4%) | 3 (2.0%) | 0.100 |
| Diabetes mellitus | 27 (3.5%) | 1 (1.9%) | 20 (3.6%) | 6 (4.1%) | 0.792 |
| Smoking | 303 (39.7%) | 19 (35.8%) | 217 (38.6%) | 67 (45.3%) | 0.283 |
| White blood cell count (10^9/L) | 12.69 (3.76) | 12.71 (3.73) | 12.53 (3.74) | 13.32 (3.83) | 0.077 |
| Hematocrit (%) | 39.24 (5.26) | 38.03 (5.52) | 39.04 (5.33) | 40.42 (4.72) | 0.004 |
| Platelet count (10^9/L) | 180.49 (69.41) | 180.42 (73.74) | 180.48 (71.81) | 180.57 (58.08) | 1.000 |
| INR | 1.10 (0.19) | 1.10 (0.18) | 1.11 (0.20) | 1.10 (0.17) | 0.915 |
| APTT (seconds) | 37.06 (7.22) | 36.76 (6.66) | 37.14 (7.47) | 36.89 (6.45) | 0.886 |
| Alanine transaminase (U/L) | 21.70 [14.00, 35.20] | 18.90 [13.80, 33.30] | 21.45 [13.72, 34.77] | 22.65 [15.95, 43.28] | 0.208 |
| Creatinine (μmol/L) | 120.71 (109.99) | 125.91 (129.13) | 122.19 (115.47) | 113.25 (76.50) | 0.638 |
| Left ventricle diameter (cm) | 46.86 (6.02) | 47.83 (6.73) | 46.73 (5.89) | 47.00 (6.22) | 0.423 |
| LVEF (%) | 65.05 (7.11) | 65.55 (5.78) | 65.00 (7.33) | 65.09 (6.70) | 0.863 |
| Time from onset to admission (hours) | 13.00 [8.00, 24.00] | 14.00 [9.00, 30.00] | 13.00 [9.00, 24.00] | 11.50 [8.00, 18.25] | 0.029 |
| Time from admission to operation (hours) | 16.00 [10.00, 28.00] | 16.00 [10.00, 23.00] | 16.00 [10.00, 28.27] | 15.67 [9.50, 24.38] | 0.695 |
| Concomitant procedures |  |  |  |  |  |
| Bentall | 52 (6.8%) | 5 (9.4%) | 33 (5.9%) | 14 (9.5%) | 0.221 |
| Aortic sinus repair | 401 (52.6%) | 23 (43.4%) | 283 (50.4%) | 95 (64.2%) | 0.004 |
| CABG | 48 (6.3%) | 2 (3.8%) | 32 (5.7%) | 14 (9.5%) | 0.177 |
| Cannulation |  |  |  |  | 0.548 |
| Axillary artery | 434 (56.9%) | 34 (64.2%) | 325 (57.8%) | 75 (50.7%) |  |
| Femoral artery | 115 (15.1%) | 7 (13.2%) | 80 (14.2%) | 28 (18.9%) |  |
| Axillary and femoral arteries | 200 (26.2%) | 11 (20.8%) | 148 (26.3%) | 41 (27.7%) |  |
| Others | 14 (1.8%) | 1 (1.9%) | 9 (1.6%) | 4 (2.7%) |  |
| Durations (minutes) |  |  |  |  |  |
| CPB | 232.58 (95.46) | 208.70 (95.37) | 222.23 (92.34) | 280.45 (92.50) | < 0.001 |
| Aorta clamping | 110.02 (54.48) | 100.19 (58.30) | 104.41 (51.76) | 134.84 (56.44) | < 0.001 |
| LBCA | 21.14 (7.75) | 20.70 (8.69) | 20.78 (7.91) | 22.66 (6.56) | 0.029 |
| Repeated CPB | 123 (16.1%) | 16 (30.2%) | 96 (17.1%) | 11 (7.4%) | < 0.001 |
| Flow of LBCA (L/min) | 1.36 (0.64) | 1.45 (0.65) | 1.39 (0.65) | 1.24 (0.55) | 0.028 |
| Nasopharyngeal temperature (°C) | 27.75 (2.03) | 28.32 (2.10) | 27.82 (2.10) | 27.29 (1.58) | 0.002 |
| Outcomes |  |  |  |  |  |
| Short–term death | 44 (5.8%) | 4 (7.5%) | 33 (5.9%) | 7 (4.7%) | 0.738 |
| Ischemic stroke | 49 (6.4%) | 2 (3.8%) | 37 (6.6%) | 10 (6.8%) | 0.765 |
| AKI | 296 (38.8%) | 18 (34.0%) | 209 (37.2%) | 69 (46.6%) | 0.084 |
| CRRT | 70 (9.2%) | 6 (11.3%) | 47 (8.4%) | 17 (11.5%) | 0.443 |
| Paraplegia | 14 (1.8%) | 0.00 (0.00) | 9 (1.6%) | 5 (3.4%) | 0.187 |
| Cardiac arrest | 41 (5.4%) | 2 (3.8%) | 30 (5.3%) | 9 (6.1%) | 0.846 |
| Tracheostomy | 25 (3.3%) | 1 (1.9%) | 18 (3.2%) | 6 (4.1%) | 0.781 |
| ICU duration (days) | 6.89 (8.49) | 5.87 (7.11) | 6.86 (8.96) | 7.39 (6.98) | 0.528 |
| CAD: coronary artery disease; INR: international normalized ratio; APTT: activated partial thromboplastin time; LVEF: left ventricular ejection fraction; CABG: coronary artery bypass grafting; CPB: cardiopulmonary bypass; LBCA: lower body circulatory arrest; AKI: acute kidney injury; CRRT: continuous renal replacement therapy; ICU: intensive care unit. | | | | | |

**Supplementary Table S5. Preoperative and intraoperative variables of matched patients grouped by nasopharyngeal rewarming rate***

| **Variables** | **All matched**  **(n = 159)** | **Nasopharyngeal rewarming rate groups** | | | **SMD** | | | **P value** |
| --- | --- | --- | --- | --- | --- | --- | --- | --- |
|  |  | **≥0.5**°**C/min**  **(n = 53)** | **0.2–0.5**°**C/min (n = 53)** | **≤0.2**°**C/min**  **(n = 53)** | **≥0.5**°**C/min vs. 0.2–0.5**°**C/min** | **≥0.5**°**C/min vs. ≤0.2**°**C/min** | **≤0.2**°**C/min vs. 0.2–0.5**°**C/min** |  |
| Male | 117 (73.6%) | 37 (69.8%) | 39 (73.6%) | 41 (77.4%) | 0.160 | 0.052 | 0.108 | 0.749 |
| Age (years) | 49.52 (11.11) | 50.32 (12.39) | 51.08 (10.36) | 47.17 (10.27) | 0.268 | 0.159 | 0.457 | 0.128 |
| Height (cm) | 166.78 (7.81) | 166.32 (7.03) | 166.79 (8.25) | 167.23 (8.21) | 0.193 | 0.027 | 0.201 | 0.564 |
| Hypertension | 117 (73.6%) | 40 (75.5%) | 42 (79.2%) | 35 (66.0%) | 0.100 | 0.105 | 0.204 | 0.632 |
| CAD | 3 (1.9%) | 2 (3.8%) | 1 (1.9%) | 0 (0.0%) | 0.214 | 0.214 | 0.000 | 0.365 |
| Previous heart surgery | 10 (6.3%) | 3 (5.7%) | 3 (5.7%) | 4 (7.5%) | 0.000 | 0.098 | 0.098 | 0.860 |
| Diabetes mellitus | 3 (1.9%) | 1 (1.9%) | 1 (1.9%) | 1 (1.9%) | 0.000 | 0.000 | 0.000 | 1.000 |
| Smoking | 60 (37.7%) | 19 (35.8%) | 20 (37.7%) | 21 (39.6%) | 0.046 | 0.046 | 0.092 | 0.910 |
| White blood cell count (10^9/L) | 12.68 (3.87) | 12.71 (3.73) | 12.66 (4.00) | 12.68 (3.95) | 0.046 | 0.106 | 0.059 | 0.886 |
| Hematocrit (%) | 38.91 (5.30) | 38.03 (5.52) | 39.14 (5.29) | 39.56 (5.05) | 0.289 | 0.040 | 0.258 | 0.351 |
| Platelet count (10^9/L) | 175.94 (62.99) | 180.42 (73.74) | 172.58 (63.21) | 174.81 (50.90) | 0.148 | 0.099 | 0.046 | 0.776 |
| INR | 1.10 (0.16) | 1.10 (0.18) | 1.11 (0.11) | 1.10 (0.17) | 0.029 | 0.003 | 0.034 | 0.985 |
| APTT (seconds) | 36.78 (6.51) | 36.76 (6.66) | 37.39 (6.58) | 36.20 (6.35) | 0.075 | 0.068 | 0.138 | 0.807 |
| Alanine transaminase (U/L) | 20.10 [14.10, 37.55] | 18.90 [13.80, 33.30] | 21.20 [15.00, 47.50] | 19.90 [15.30, 34.60] | 0.111 | 0.100 | 0.021 | 0.655 |
| Creatinine (μmol/L) | 119.93 (103.12) | 125.91 (129.13) | 123.75 (103.77) | 110.15 (68.67) | 0.217 | 0.010 | 0.285 | 0.508 |
| Left ventricle diameter (cm) | 47.88 (6.32) | 47.83 (6.73) | 47.15 (6.12) | 48.66 (6.12) | 0.117 | 0.084 | 0.224 | 0.624 |
| LVEF (%) | 65.86 (6.65) | 65.55 (5.78) | 65.75 (6.90) | 66.26 (7.28) | 0.063 | 0.053 | 0.014 | 0.955 |
| Time from onset to admission (hours) | 14.00 [9.00, 29.50] | 14.00 [9.00, 30.00] | 12.00 [8.00, 30.00] | 16.00 [10.00, 26.00] | 0.098 | 0.080 | 0.020 | 0.778 |
| Time from admission to operation (hours) | 15.33 [9.00, 22.50] | 16.00 [10.00, 23.00] | 15.67 [7.00, 25.50] | 13.84 [9.25, 20.00] | 0.403 | 0.089 | 0.325 | 0.307 |
| Concomitant procedures |  |  |  |  |  |  |  |  |
| Bentall | 18 (11.3%) | 5 (9.4%) | 3 (5.7%) | 10 (18.9%) | 0.143 | 0.084 | 0.225 | 0.550 |
| Aortic sinus repair | 82 (51.6%) | 23 (43.4%) | 32 (60.4%) | 27 (50.9%) | 0.228 | 0.228 | 0.000 | 0.465 |
| CABG | 6 (3.8%) | 2 (3.8%) | 2 (3.8%) | 2 (3.8%) | 0.125 | 0.125 | 0.000 | 0.812 |
| Cannulation |  |  |  |  | 0.058 | 0.214 | 0.214 | 0.977 |
| Axillary artery | 98 (61.6%) | 34 (64.2%) | 32 (60.4%) | 32 (60.4%) |  |  |  |  |
| Femoral artery | 25 (15.7%) | 7 (13.2%) | 10 (18.9%) | 8 (15.1%) |  |  |  |  |
| Axillary and femoral arteries | 32 (20.1%) | 11 (20.8%) | 10 (18.9%) | 11 (20.8%) |  |  |  |  |
| Others | 4 (2.5%) | 1 (1.9%) | 1 (1.9%) | 2 (3.8%) |  |  |  |  |
| Durations (minutes) |  |  |  |  |  |  |  |  |
| CPB | 212.60 (80.42) | 208.70 (95.37) | 222.89 (68.62) | 206.23 (75.40) | 0.255 | 0.053 | 0.359 | 0.272 |
| Aorta clamping | 104.26 (50.60) | 100.19 (58.30) | 110.04 (44.53) | 102.57 (48.41) | 0.237 | 0.015 | 0.296 | 0.383 |
| LBCA | 21.52 (8.29) | 20.70 (8.69) | 22.55 (7.20) | 21.30 (8.93) | 0.144 | 0.032 | 0.191 | 0.656 |
| Repeated CPB | 29 (18.2%) | 16 (30.2%) | 4 (7.5%) | 9 (17.0%) | 0.000 | 0.206 | 0.206 | 0.560 |
| Flow of LBCA (L/min) | 1.42 (0.69) | 1.45 (0.65) | 1.32 (0.59) | 1.50 (0.80) | 0.246 | 0.065 | 0.316 | 0.282 |
| Nasopharyngeal temperature (°C) | 28.15 (2.06) | 28.32 (2.10) | 27.78 (1.93) | 28.35 (2.13) | 0.189 | 0.199 | 0.410 | 0.192 |
| *: A 1:1:1 ratio without replacement was used in propensity score matching.  SMD: standard mean difference; CAD: coronary artery disease; INR: international normalized ratio; APTT: activated partial thromboplastin time; LVEF: left ventricular ejection fraction; CABG: coronary artery bypass grafting; CPB: cardiopulmonary bypass; LBCA: lower body circulatory arrest. | | | | | | | | |

**Supplementary Table S6. Outcomes of matched patients grouped nasopharyngeal rewarming rate**

| **Variables** | **All matched (n = 159)** | **Nasopharyngeal rewarming rate groups** | | | **Overall P value** | **Pairwise Holm–adjusted**  **P value** | | |
| --- | --- | --- | --- | --- | --- | --- | --- | --- |
|  |  | **≥0.5**°**C/min (n = 53)** | **0.2–0.5**°**C/min (n = 53)** | **≤0.2**°**C/min (n = 53)** |  | **≥0.5**°**C/min vs. 0.2–0.5**°**C/min** | **≥0.5**°**C/min vs. ≤0.2**°**C/min** | **≤0.2**°**C/min vs. 0.2–0.5**°**C/min** |
| Short–term death | 13 (8.2%) | 4 (7.5%) | 2 (3.8%) | 7 (13.2%) | 0.204 | 0.490 | 1.000 | 1.000 |
| Ischemic stroke | 8 (5.0%) | 2 (3.8%) | 3 (5.7%) | 3 (5.7%) | 0.877 | 0.988 | 1.000 | 1.000 |
| AKI | 55 (34.6%) | 18 (34.0%) | 18 (34.0%) | 19 (35.8%) | 0.973 | 1.000 | 1.000 | 1.000 |
| CRRT | 13 (8.2%) | 6 (11.3%) | 4 (7.5%) | 3 (5.7%) | 0.556 | 1.000 | 1.000 | 1.000 |
| Paraplegia | 0 (0.0%) | 0 (0.0%) | 0 (0.0%) | 0 (0.0%) | 1.000 | 1.000 | 1.000 | 1.000 |
| Cardiac arrest | 7 (4.4%) | 2 (3.8%) | 1 (1.9%) | 4 (7.5%) | 0.351 | 1.000 | 1.000 | 1.000 |
| Tracheostomy | 3 (1.9%) | 1 (1.9%) | 2 (3.8%) | 0 (0.0%) | 0.361 | 1.000 | 1.000 | 1.000 |
| ICU duration (days) | 5.46 (5.51) | 5.46 (5.51) | 5.87 (7.11) | 5.60 (5.34) | 0.652 | 1.000 | 1.000 | 1.000 |
| AKI: acute kidney injury; CRRT: continuous renal replacement therapy; ICU: intensive care unit. | | | | | | | | |


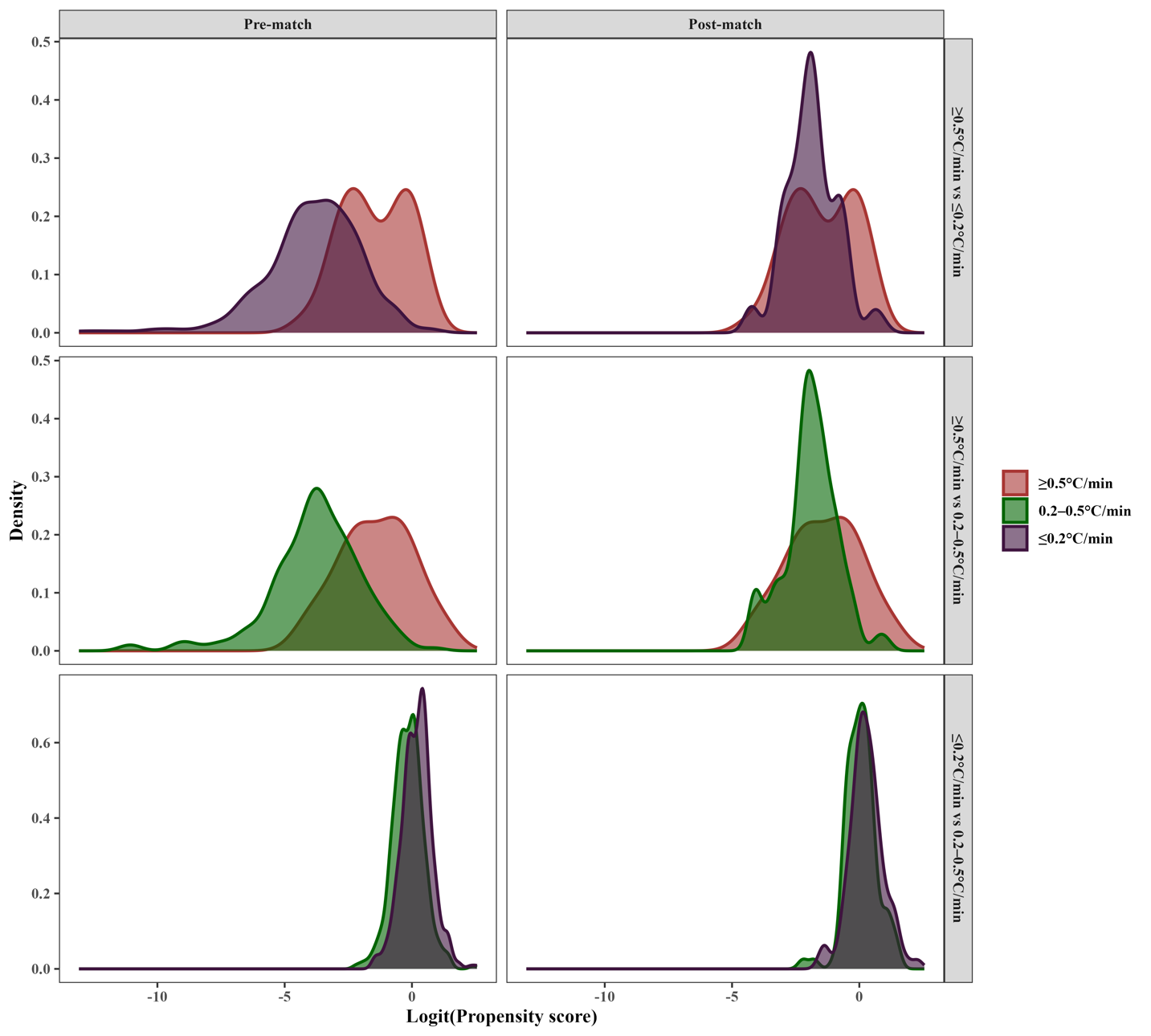
**Supplementary Figure S1:** Propensity–score distribution plots between matched groups.

**
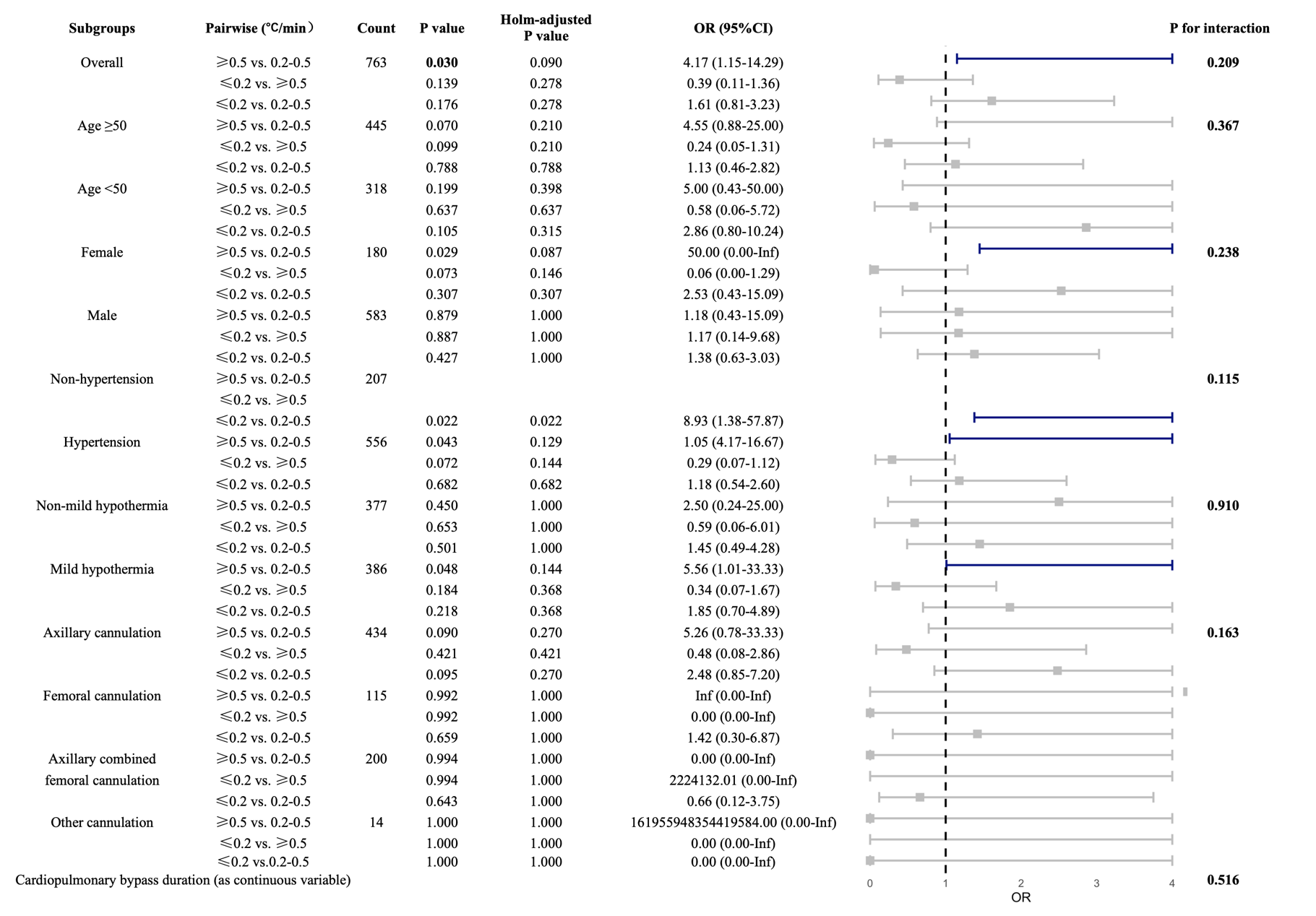
Supplementary Figure S2:** Subgroup analyses on effect modification of fast bladder rewarming on short–term death (no events/no exposure in the pairwise comparison of Non–hypertension subgroup: ≥0.5 vs. 0.2–0.5, and ≤0.2 vs. ≥0.5; CPB: cardiopulmonary bypass).
